# Machine Learning Models on ADC Features to Assess Brain Changes of Children With Pierre Robin Sequence

**DOI:** 10.3389/fneur.2021.580440

**Published:** 2021-03-04

**Authors:** Ying Wang, Feng Yang, Meijiao Zhu, Ming Yang

**Affiliations:** Department of Radiology, Children's Hospital of Nanjing Medical University, Nanjing, China

**Keywords:** ADC features, machine learning, Pierre Robin sequence, brain changes, MRI

## Abstract

In order to evaluate brain changes in young children with Pierre Robin sequence (PRs) using machine learning based on apparent diffusion coefficient (ADC) features, we retrospectively enrolled a total of 60 cases (42 in the training dataset and 18 in the testing dataset) which included 30 PRs and 30 controls from the Children's Hospital Affiliated to the Nanjing Medical University from January 2017–December 2019. There were 21 and nine PRs cases in each dataset, with the remainder belonging to the control group in the same age range. A total of 105 ADC features were extracted from magnetic resonance imaging (MRI) data. Features were pruned using least absolute shrinkage and selection operator (LASSO) regression and seven ADC features were developed as the optimal signatures for training machine learning models. Support vector machine (SVM) achieved an area under the receiver operating characteristic curve (AUC) of 0.99 for the training set and 0.85 for the testing set. The AUC of the multivariable logistic regression (MLR) and the AdaBoost for the training and validation dataset were 0.98/0.84 and 0.94/0.69, respectively. Based on the ADC features, the two groups of cases (i.e., the PRs group and the control group) could be well-distinguished by the machine learning models, indicating that there is a significant difference in brain development between children with PRs and normal controls.

## Introduction

Pierre Robin sequence (PRs) is a congenital condition characterized by an abnormal development of craniofacial features. The incidence of PRs is as high as 1/8,500–1/14,000 (1). The characteristics of PRs include: micrognathia, glossoptosis, and cleft palate. These defects can lead to airway obstruction and feeding difficulties (2), even life-threatening obstructive apnea and obstructive sleep apnea in neonates (3). Moreover, these structural abnormalities seriously affect the growth and development of children (4), placing a burden on their families as well as society. At present, most PRs research has focused on its pathogenesis (5), prenatal diagnosis (6), surgical treatment plans, and post-operative nursing and feeding (7), as well as cognitive and neurological development (8). Little attention has been paid to the quantitative evaluation of brain development based on MRI in children with PRs, which limits the ability to fully understand the inherent neurological function impairment.

Diffusion-weighted imaging (DWI) has features such as greater sensitivity for ischemia and water molecule diffusion compared to other MR imaging techniques. However, a strong T2 shine-through effect limits its application in the quantitative evaluation of neonates. ADC maps have been proposed to evaluate neonatal hypoxic-ischemic brain injury, tumor and brain development, etc. (9, 10), which has been used as an important imaging modality. Therefore, the ADC features are an important sequence for exploring development in children. Previous studies have revealed that pre-existing hypoxia may cause damage to the brain. However, some hypoxic damage does not typically lead to structural changes that cannot be detected by a radiologist based on conventional MRI.

Machine learning, however, can uncover important information that is undetected by routine clinical medical imaging, thus revealing potential biological information (11–13). Given its compelling advantages in the implementation of classification and prediction (14, 15), machine learning has become a common method of scientific research (16, 17). In this study, we analyzed ADC features combined with several machine learning models to effectively evaluate brain development differences between children with PRs and normal controls as well as the validity of the model. To the best of our knowledge, this is the first study to quantitatively evaluate the brain development of PRs based on MRI with machine learning models.

Our research group focuses on studying the brain development of congenital heart disease (CHD) and cerebral palsy (CP) based on ADC. To date, there are few other studies on this subject, and most previous research on ADC has focused on adults or positive lesions. In these instances ADC is only used for diagnosis, without extracting its inherent imaging features. Based on this, our research combines them and can also reveal methodological advantages to studying the brain development of children with PRs. We think that this study could also be applied to conventional “lesion negative” MRIs in the future, such as pediatric epilepsy, Autism, and Attention Deficit Hyperactivity Disorder (ADHD).

## Materials and Methods

### Enrolled Patients

**Figure 1:**
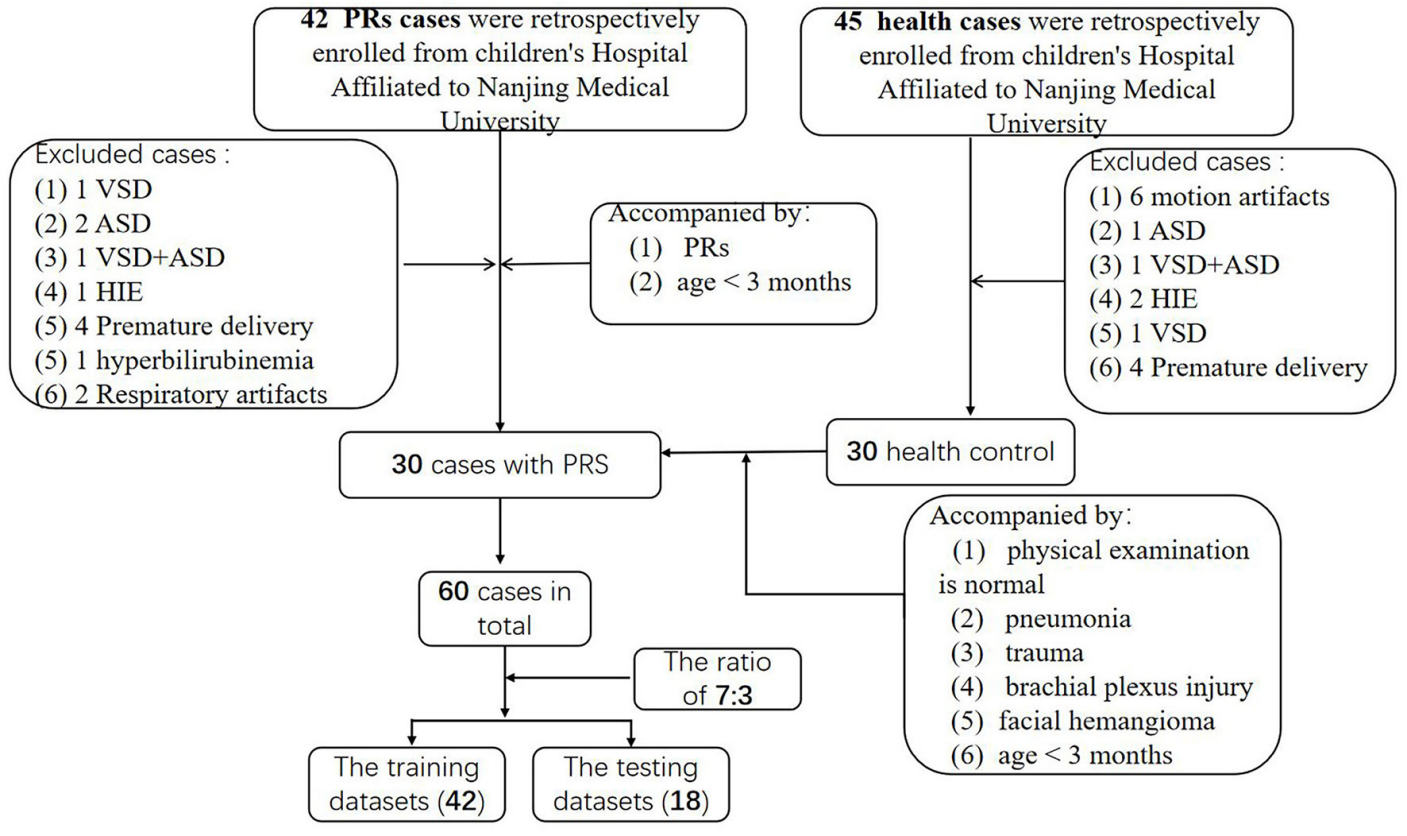
Flow diagram of enrolled patients. HIE, neonatal hypoxic-ischemic encephalopathy; VSD, ventricular septal defect; ASD, atrial septal defect.

A retrospective comparative dataset study was conducted, ultimately consisting of 30 children diagnosed with PRs and 30 healthy controls (Figure 1). A ratio of 7:3 was used to randomly assign these cases to the training and validation datasets (18). Therefore, 42 cases were assigned to the training dataset, of which, 21 were PRs cases. The remaining 18 cases, including nince cases in the PRs group, were assigned to the testing dataset. Patients in the PRs group were hospitalized in the plastic surgery department of the Children's Hospital of Nanjing Medical University from January 2017 to December 2019 and were clinically diagnosed with PRs. The mean age of the PRs group was 39.3 ± 19 days (range: 3–84 days) and consisted of 12 males and 18 females. The inclusive criteria of this group were: (1) clinically-confirmed PRs; (2) age <3 months; and (3) no clinical operation. Exclusion criteria: the images included respiratory motion artifacts, brain malformation, neonatal hypoxic-ischemic encephalopathy (HIE), hyperbilirubinemia, or other encephalopathy, such as ventricular septal defect (VSD), atrial septal defect (ASD).

The control group, which had a mean age of 45.2 ± 18 days (range: 7–74 days), consisted of 12 males and 18 females. There were 45 cases in the control group initially. After screening, 30 cases were selected. The inclusive criteria of this group were: (1) normal brain MRI of brain, as confirmed by two experienced pediatric radiologists. The admission history of these patients included pneumonia, trauma, brachial plexus injury, and facial hemangioma; and (2) age <3 months; (3) No definite brain developmental diseases were found during follow-up; (4) MRI images meet the diagnostic requirements (no obvious motion artifacts). The exclusion criteria include: (1) suspected or confirmed neurologic disease in labor; (2) children with known genetic abnormalities (e.g., Down syndrome) or other congenital diseases (e.g., congenital heart disease); and (3) other serious diseases that may have substantial effects on the brain; (4) MRI images cannot be diagnosed (there are obvious motion artifacts).

This study was approved by the hospital ethics committee. Before the MRI scan, parents were informed of the precautions before the examination, read and signed the informed consent form, and explained the examination method and process to children. As a retrospective study, each participant was followed up by telephone, informed of the significance of participating in this study, and agreed to participate. On follow up each patient was informed of the significance of the study for the child's future brain development, and their consent was obtained.

### Imaging Protocol

MR imaging of the brain was performed using a 3-Tesla (T) whole-body clinical MRI scanner (Ingenia; Philips Healthcare, Best, The Netherlands), with a digital head coil. The patients were sedated with 5% chloral hydrate (1 mL/kg) 20 min before the scan, and the MR examination was performed while the patients were asleep. An SE-EPI sequence was used in the DWI scan, and axial DWI sequences were generated at b values of 0 and 1,000 s/mm^2^ at TR/TE = 2,408/82 ms. The layer thickness was 4-mm, and the layer spacing was 1-mm. The field of view = 180 × 180 mm. There were three orthogonal diffusion gradient directions and two excitations. The phase encoding direction was anterior to posterior (AP).

### Image Acquisition and Retrieval

The ADC images were exported from a PACS workstation in DICOM format, and single ADC map slices were selected at the level of the thalamus/basal ganglia, the most sensitive injury site for children, as previously illustrated (19).

### Feature Extraction and Pre-processing

A single-layer ADC map of the lateral ventricle at the anterior and posterior horn level was selected using the *ImgJ* software. Single ADC images were imported into the image processing software (MRIcron), after which the complete brain area (remove the extracerebral space) was sketched (with the extracerebral space removed) as the region of interest (ROI), and saved in NIFTI format. In addition, ADC feature extraction was implemented using an open source Python software package (www.python.org). Importing Feature Analysis module as well as library functions included numpy, pre-process to realize the feature extraction in the python code (the original code was provided in the Supplementary Materials with PDF form). A total of 104 features were extracted from the ADC images, including density histograms (features 1–27), textures (28–33), and wavelets (34–104). In order to guarantee the normalization of the results, z-score normalization was utilized to pre-process these images and data. After z-score normalization, 103 features were retained (the 33rd feature was excluded because it feature represents the correlation in the texture feature. All values in the two groups were 1, which was meaningless for classification).

## Data Analysis

### Dimension Reduction and Feature Selection

To avoid dimensional disaster, enhance features robustness, and prevent the model from overfitting, it was necessary to reduce the dimensions of the features. The dimensionality reduction process was roughly divided into the following steps: first, the features were tested for normality and homogeneity of variance, after which the *p* > 0.05 results were preserved. Next, either the independent sample *t*-test or rank sum test (Mann Whitney *U*-test) was used based on the feature distribution. Finally, LASSO regression was performed for dimensionality reduction and feature selection. Parameter adjustment could then improve the prediction accuracy and credibility of the machine learning model. After optimization, the minimum deviance criterion was implemented in our study to adjust the regularization parameter (λ), and 10-fold cross-validation was used to enhance feature robustness. Because principal component analysis (PCA) is a type of unsupervised learning, LASSO is more universally applied than either PCA or SVD (singular value decomposition), and the model complexity can be adjusted by the parameter λ (lambda) to avoid overfitting (20).

### Machine Learning Model Construction

**Figure 2:**
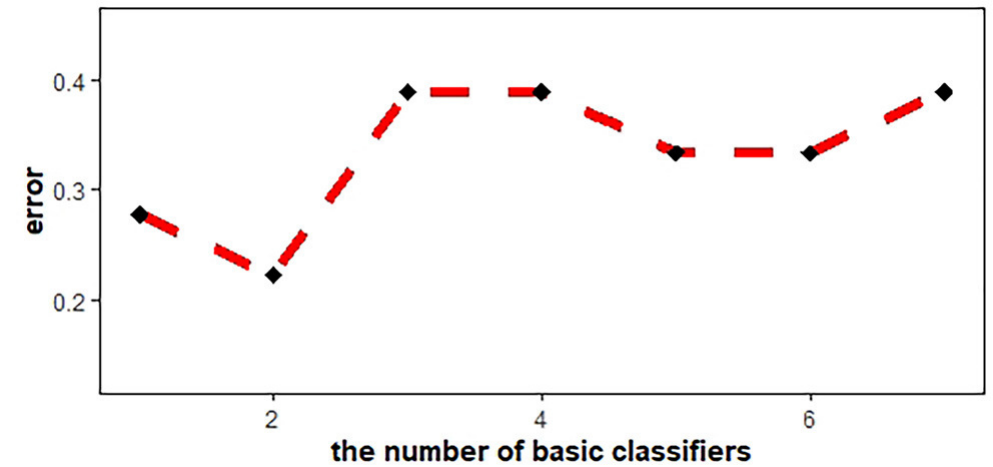
AdaBoost error line chart. When the number of basic classifiers was 2, the error reached the minimum value.

Machine learning models, including multivariate logistic regression (MLR), support vector machine (SVM), and AdaBoost, were introduced to investigate whether the ADC features of the two groups (PRs patients and healthy controls) were linearly separable, which would provide conclusive evidence of the brain development differences. SVM with Gaussian kernel function was constructed as the non-linear classifier. The ranges of the optimal kernel function γ(gamma) and hyperparameter C(cost) were 10^∧^(-3:0) and 10^∧^(0:3), respectively. These best performing signatures were realized by10-fold cross-validation, which enhanced model robustness. Logistic regression was then used with backward elimination to construct a linear classifier model. Newton's method was employed to determine the maximum point of the logistic log-likelihood function. The number of iterations was set to 10. In addition, the AdaBoost algorithm was introduced. By plotting the error line chart, it was revealed that when the number of weak classifiers was two, the minimum error value was achieved (Figure 2). Finally, the AUC curve, classification accuracy, positive predictive value (PPV), negative predictive value (NPV), sensitivity, and specificity were also calculated in order to assess the discrimination capability.

### Statistics

SPSS25 statistical software was utilized. The *t*-test and chi-square-test were used to analyze the age and gender to ensure that there was no difference between the two groups.

R language (version 3.4.1, https://www.r-project.org/) was employed to analyze the ADC characteristic values. Zero-mean normalization was applied to eliminate the impacts of dimensions and value range differences, and 70% of the cases were randomly selected as the training dataset without replacement (18). Univariate analysis was performed on the values of the training dataset. Independent sample *t*-tests were implemented on values that satisfied normality and variance uniformity. The remainder were subjected to the Wilcoxon rank sum test. LASSO regression and coefficient curves were generated using the “*glmne*t” and “*ggplot2*” software packages. The SVM classifier was executed by the “*e1071*” package, while the “*adabag*” package was utilized for the AdaBoost algorithm, and the “caret,” “lattice,” and “for each” packages were also applied. ROC curves were realized with the “*pROC*” package.

## Results

### Features Change Between Two Groups

For ADC histogram characteristics, except for *ADCmax, Skewness, Kurtosis, Entropy*, and *variance*, the ADC histogram characteristics of the PRs group are lower than the normal group, and the differences are statistically significant (*P* < 0.05). For texture features, the values of *contrast* and *dissimilarity* in the PRs group are lower than those in the normal group. However, for the values of *homogeneity, ASM*, and *energy*, the PRs are higher than the normal group, and the differences are statistically significant. [See the Supplementary Material in the table, which lists all ADC characteristic values (histogram, texture, wavelet) and the *t*-value and *p*-value after *t*-test.]

### Dimension Reduction and Feature Selection

**Figure 3:**
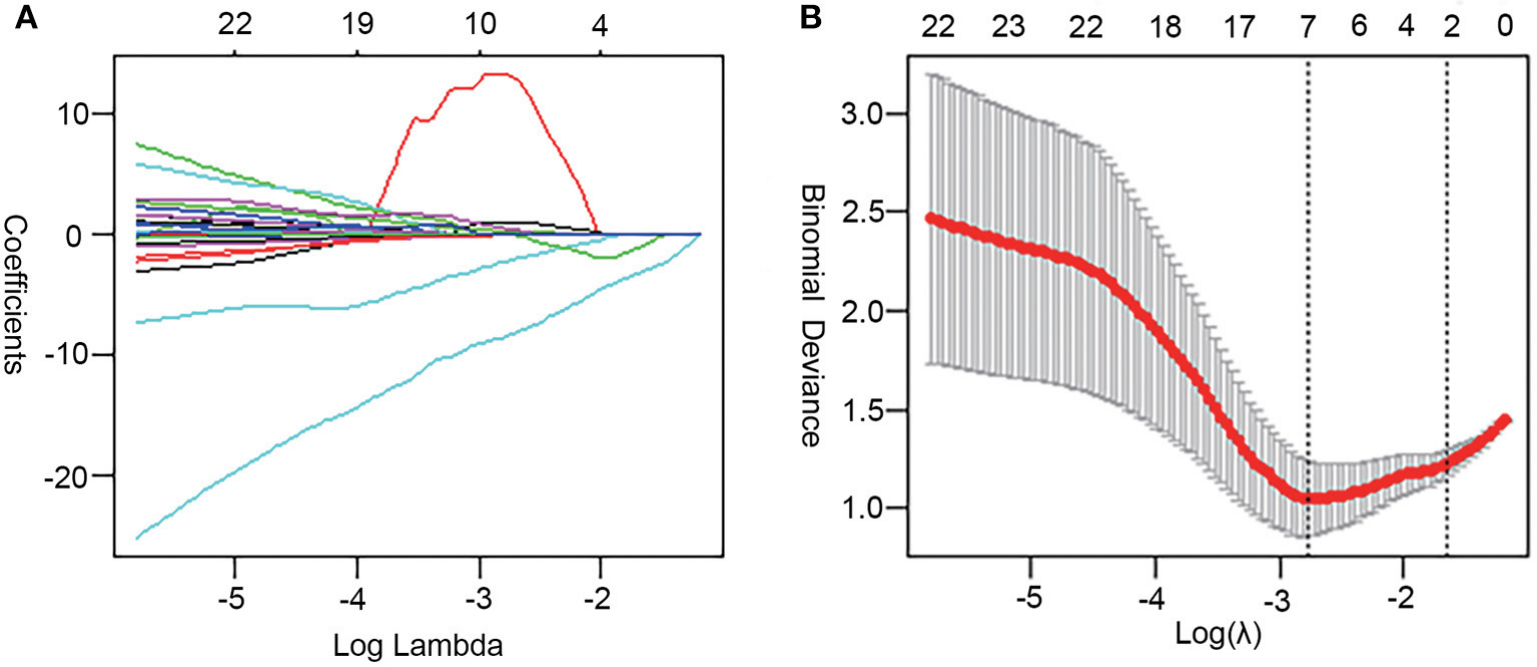
Feature selection by LASSO regression. **(A)** LASSO coefficient sketch of 99 features. With increasing lambda, the coefficient of unimportant features gradually tended to 0. **(B)** The deviance profile was made by adjusting the optimal regularization parameters (λ) based on 10-fold cross-validation. The left dashed line delineates the minimum norm and the right indicates 1-standard error norm (1-SE). This study utilized the minimum norm.

A total of 39 features were preserved using a Gaussian distribution with variance homogeneity, all of which were performed using independent sample *t*-tests. Of the remaining 65 features, 60 features were found to be significant by mean of the Mann-Whitney *U*-test. Therefore, LASSO regression was performed on these 99 features (i.e., 39 that passed the *t*-test and that 60 passed the rank sum-test). Furthermore, the optimal signatures were revealed when the binomial deviance achieved its minimum values. Finally, the seven features (the 29th, 30th, 39th, 56th, 62th, 92th, and 96th) were pruned using LASSO regression, with the optimal regularization parameter λ of 0.0627 under the minimum norm tuned by 10-fold cross validation (Figure 3).

### Performance of machine learning

The SVM was verified to exhibit the most optimal performance for the training dataset, with an AUC of 0.998 (95% CI: 0.991–1), a discrimination accuracy of 98% by the tuned best kernel function (Gaussian kernel γ) of 0.1, and a hyperparameter (C) of 10. The AUC of the multivariate logistic regression model was confirmed to be 0.980 (95% CI: 0.942–1) for the training dataset, the discrimination accuracy of the MLR was 93%. The performance of the AdaBoost algorithm was confirmed to have an AUC of 0.94 (95% CI: 0.872–1) and a discrimination accuracy of 86.0% for the training dataset. The testing dataset also achieved good performance using all of the machine learning models, although not as good as the training group (Table 1).

**Table 1:** Performance of machine learning models for training datasets and testing datasets.

	**AUC**	**Accuracy**	**Sensitivity (%)**	**Specificity (%)**	**PPV (%)**	**NPV (%)**	
The training datasets	SVM	99.8	97.6	100	95.2	95.5	100
	MLR	98.0	92.9	95.2	90.5	90.9	95.0
	ADA	94.0	85.7	90.5	81.0	82.6	89.5
The testing datasets	SVM	85.2	77.8	66.7	88.9	85.7	72.7
	MLR	84.0	72.2	66.7	77.8	75.0	70.0
	ADA	68.5	66.7	66.7	66.7	66.7	66.7

*SVM, support vector machine; MLR, multivariate logistic regression; ADA, AdaBoost; PPV, positive predictive value; NPV, negative predictive value*.

**Figure 4:**
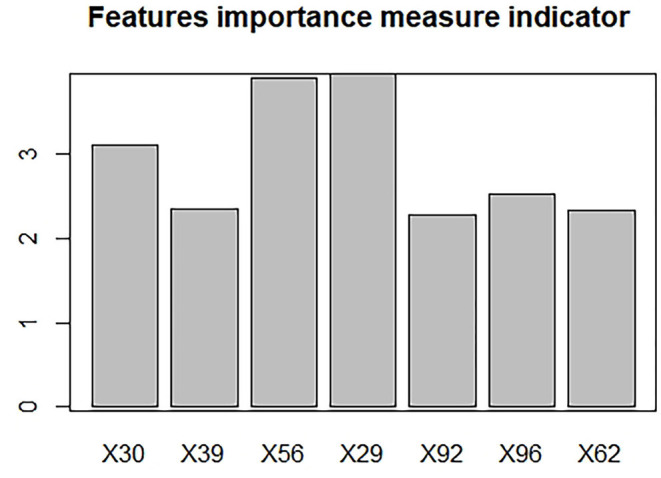
The features importance measurement indicator.

In the ML (machine learning) models, the features selected after dimension reduction all played an important role in the discrimination of PRs from controls. Among them, the 29th represents dissimilarity, the 30th represents homogeneity, the 39th and 56th represents the first order wavelet, the 62nd represents the second order wavelet, and the 92nd and 96th represent the third order wavelet. The histogram was made to show the importance about each feature (Figure 4). For the histogram, it can be seen that the 29th, 30th, and 56th are the top three important features.

## Discussion

At present, ADC features have been widely used in tumor research and show good repeatability (21). However, this method is not widely used in neonatal non-tumor lesions. Little attention has been paid to ADC features of MRI based on machine learning on infant's brain changes for previous studies. Previous studies have proved that diffusion-weighted imaging (DWI) is most sensitive for water molecules, ischemia, and hypoxia, and the ADC map, as post-processing sequence of DWI, is especially suitable for infant brain development researches, such as HIE (Neonatal hypoxic–ischemic encephalopathy), PLIC (the posterior limb of the internal capsule) (22). There are also a few related studies on the brain development of PRs. We found that the ADC histogram characteristics of the PRs group were relatively lower than that of the normal group, which may be related to hypoxia and abnormal brain development (19, 23). The texture characteristics and wavelet characteristics were also significantly different in our two groups.

As an effective tool for data classification and prediction, machine learning has been widely used in medical imaging, especially in the research of radiomics. Based on features of the ADC, machine learning was utilized, which was simpler and more accurate than traditional statistical methods (12, 24), especially for cases involving multidimensional features. In this study, the parameters were optimized to ensure the accuracy and credibility of the research results. LASSO regression was employed for feature selection, ensuring that the important and independent features conformed to the models. After 10-fold cross-validation, the robustness of the features was ensured.

In our research, the SVM and MLS models were found to exhibit better classification accuracy and performance than the AdaBoost models, which was consistent with previous research on the performance of machine learning models (25). The SVM model achieved the most optimal performance with an AUC of 0.998 and a discrimination accuracy of 98.0% for the training dataset. An AUC of MLR was confirmed to 0.980 and a discrimination accuracy of 93.0% for the training dataset. These good performance results revealed that the two groups of cases (PRs patients and normal controls) were clearly distinguished by the machine learning models, indicating that there are significant differences in the brain development of these children. In total, the three machine learning models all achieved a good overall performance. For the testing cohort, the SVM model also achieved the most optimal performance with an AUC of 0.852, and good discrimination accuracy of 77.8%, and a specificity of 88.9%. An AUC of MLR was confirmed to 0.840, and a discrimination accuracy of 72.2% and specificity of 77.8% for the testing dataset. However, the AUC, sensitivity, and specificity of the AdaBoost in the testing dataset relative dropper are 68.5, 66.7, and 66.7% (26–28).

The reasons for the relatively weak results of the validation set may be as follows:

- As infants are still young, it is more difficult to perform magnetic resonance examinations. Therefore, it is difficult to collect the normal control group. This leads to a small amount of data being entered into the group, resulting in relatively weak results for the validation set.
- When using cross-validation to divide the data set, the parameter setting of the random seed makes the random allocation of data biased.

At the same time, this research had some limitations. Firstly, due to limited resources, the data were acquired from a single medical center and the same type of MRI scanner. It will be more convincing to acquire images from multiple institutions or MRI models. Multivariate clinical case information would also be an improvement. Secondly, to facilitate clinical operations, only one level of ADC map was evaluated, which may not be comprehensive. Thirdly, the control group may have included children with abnormal brain development simply due to a lack of totally healthy volunteers. Fourthly, no scale was used to evaluate the development of neurological function in PRs patients. Finally, although this study achieved good discrimination based on ADC features, there were no specific predicted results to explain the prediction trends due to a lack of follow up research data.

In conclusion, in this study, we focused on ADC features in the pediatric brain, confirming that the two groups of cases, which included PRs patients and normal controls, were well-distinguished by machine learning models, indicating that there was significant brain development abnormality in PRs children. Compared with other statistical methods, machine learning models are simple and clear, with effective operability and high accuracy.

## Data Availability Statement

The datasets generated in this study can be found in online repositories. The names of the repository/repositories and accession number(s) can be found in the article/Supplementary Material.

## Ethics Statement

The studies involving human participants were reviewed and approved by Children's Hospital of Nanjing Medical University Ethics Committee. Written informed consent to participate in this study was provided by the participants' legal guardian/next of kin.

## Author Contributions

YW prepared the data and drafted the medical sections of the manuscript. FY analyzed the data using machine learning and drafted the manuscript. MZ extracted data features. MY guided and revised the manuscript throughout. All authors contributed to manuscript development, read, and approved the final manuscript.

## Conflict of Interest

The authors declare that the research was conducted in the absence of any commercial or financial relationships that could be construed as a potential conflict of interest.
