# Supplementary material for: Machine Learning Models on ADC Features to Assess Brain Changes of Children With Pierre Robin Sequence
Source: Front Neurol. 2021 Mar 4;12:580440. doi: 10.3389/fneur.2021.580440 (PMC7969993; doi:10.3389/fneur.2021.580440)
Supplement: Supplementary file 1 [file Table_1.DOCX]

|  | PRs group | Normal control | T value | P value |
| --- | --- | --- | --- | --- |
| ADC_mean_ | 0.88±0.29 | 1.04±0.15 | 2.78 | 0.007 |
| ADC_min_ | 0.72±0.57 | 0.93±0.52 | 1.46 | 0.149 |
| ADC_max_ | 1±0.02 | 0.99±0.01 | -2.41 | 0.019 |
| Skewness | 0.95±0.69 | 0.64±0.44 | -2.12 | 0.038 |
| Kurtosis | 0.73±1.09 | 0.16±0.51 | -2.61 | 0.012 |
| Entropy | 1±0.05 | 0.99±0.03 | -0.82 | 0.414 |
| Mode_count | 0.49±0.43 | 0.67±1.06 | 0.87 | 0.391 |
| Mode_value | 0.88±0.33 | 1.04±0.12 | 2.53 | 0.014 |
| variance | 1.04±0.47 | 0.8±0.2 | -2.59 | 0.012 |
| Percentile_10 | 0.84±0.36 | 1.05±0.19 | 2.83 | 0.006 |
| Percentile_20 | 0.85±0.35 | 1.05±0.18 | 2.83 | 0.006 |
| Percentile_30 | 0.85±0.34 | 1.05±0.18 | 2.84 | 0.006 |
| Percentile_40 | 0.86±0.33 | 1.05±0.18 | 2.81 | 0.007 |
| Percentile_50 | 0.86±0.32 | 1.05±0.18 | 2.77 | 0.007 |
| Percentile_60 | 0.87±0.32 | 1.05±0.17 | 2.74 | 0.008 |
| Percentile_70 | 0.87±0.31 | 1.04±0.16 | 2.68 | 0.010 |
| Percentile_80 | 0.89±0.29 | 1.04±0.14 | 2.55 | 0.013 |
| Percentile_90 | 0.9±0.23 | 1.04±0.12 | 2.86 | 0.006 |
| Percentile_15 | 0.84±0.35 | 1.05±0.19 | 2.84 | 0.006 |
| Percentile_25 | 0.85±0.34 | 1.05±0.18 | 2.83 | 0.006 |
| Percentile_35 | 0.85±0.33 | 1.05±0.18 | 2.81 | 0.007 |
| Percentile_45 | 0.86±0.33 | 1.05±0.18 | 2.80 | 0.007 |
| Percentile_55 | 0.86±0.32 | 1.05±0.17 | 2.76 | 0.008 |
| Percentile_65 | 0.87±0.31 | 1.04±0.16 | 2.71 | 0.009 |
| Percentile_75 | 0.88±0.3 | 1.04±0.15 | 2.63 | 0.011 |
| Percentile_85 | 0.89±0.27 | 1.04±0.14 | 2.55 | 0.014 |
| Percentile_95 | 0.95±0.16 | 1.02±0.08 | 2.33 | 0.023 |
| contrast | 0.85±0.28 | 1.07±0.17 | 3.62 | 0.001 |
| dissimilarity | 0.93±0.11 | 1.04±0.07 | 4.75 | 0.000 |
| homogeneity | 1±0.01 | 0.98±0.02 | -3.79 | 0.000 |
| ASM | 1.01±0.13 | 0.95±0.15 | -1.80 | 0.077 |
| energy | 1.01±0.07 | 0.97±0.07 | -1.84 | 0.071 |
| First order wavelet | | | | |
| 1/2LL_mean_ | 0.87±0.28 | 1.06±0.17 | 3.16 | 0.002 |
| 1/2LH_mean_ | -0.26±1.05 | -0.27±0.89 | -0.04 | 0.968 |
| 1/2HL_mean_ | -0.16±1.16 | 0.2±0.78 | 1.44 | 0.157 |
| 1/2LL_standard_ | 1.03±0.33 | 0.88±0.16 | -2.31 | 0.024 |
| 1/2LH_standard_ | 1.01±0.29 | 0.91±0.22 | -1.44 | 0.155 |
| 1/2HL_standard_ | 0.92±0.24 | 1.01±0.24 | 1.45 | 0.152 |
| 1/4LL_mean_ | 0.88±0.29 | 1.05±0.15 | 2.83 | 0.006 |
| 1/4LH_mean_ | -0.1±0.9 | -0.33±1.05 | -0.90 | 0.372 |
| 1/4HL_mean_ | 0±1.13 | 0.03±0.87 | 0.11 | 0.913 |
| 1/4LL_standard_ | 1.04±0.29 | 0.89±0.13 | -2.59 | 0.012 |
| 1/4LH_standard_ | 0.96±0.21 | 0.98±0.2 | 0.49 | 0.625 |
| 1/4HL_standard_ | 0.95±0.22 | 1±0.18 | 0.96 | 0.340 |
| 1/8LL_mean_ | 0.87±0.29 | 1.05±0.16 | 3.04 | 0.004 |
| 1/8LH_mean_ | -0.48±0.9 | -0.73±0.66 | -1.24 | 0.221 |
| 1/8HL_mean_ | -0.18±1 | 0.02±1 | 0.79 | 0.431 |
| 1/8LL_standard_ | 0.98±0.18 | 0.98±0.15 | -0.09 | 0.926 |
| 1/8LH_standard_ | 0.92±0.14 | 1.04±0.15 | 3.05 | 0.003 |
| 1/8HL_standard_ | 0.94±0.14 | 1.02±0.12 | 2.42 | 0.019 |
| 1/16LL_mean_ | 0.87±0.28 | 1.05±0.18 | 3.10 | 0.003 |
| 1/16LH_mean_ | -0.09±1.17 | 0.02±0.81 | 0.41 | 0.681 |
| 1/16HL_mean_ | -0.29±0.89 | -0.52±0.94 | -1.00 | 0.322 |
| 1/16LL_standard_ | 0.91±0.24 | 1.03±0.13 | 2.46 | 0.017 |
| 1/16LH_standard_ | 0.9±0.14 | 1.06±0.1 | 4.99 | 0.000 |
| 1/16HL_standard_ | 0.91±0.17 | 1.05±0.13 | 3.41 | 0.001 |
| Second order wavelet | | | | |
| 1/2LL_mean_ | 0.86±0.28 | 1.06±0.17 | 3.18 | 0.002 |
| 1/2LH_mean_ | 0.01±1.2 | -0.05±0.78 | -0.25 | 0.806 |
| 1/2HL_mean_ | 0.15±1.2 | 0.17±0.74 | 0.07 | 0.941 |
| 1/2LL_standard_ | 1.03±0.35 | 0.87±0.17 | -2.31 | 0.025 |
| 1/2LH_standard_ | 1.06±0.34 | 0.84±0.2 | -3.02 | 0.004 |
| 1/2HL_standard_ | 0.97±0.28 | 0.93±0.3 | -0.47 | 0.639 |
| 1/4LL_mean_ | 0.88±0.29 | 1.04±0.15 | 2.78 | 0.007 |
| 1/4LH_mean_ | -0.03±1.19 | -0.09±0.78 | -0.21 | 0.833 |
| 1/4HL_mean_ | -0.44±0.92 | 0.04±0.99 | 1.93 | 0.059 |
| 1/4LL_standard_ | 1.04±0.29 | 0.88±0.14 | -2.68 | 0.010 |
| 1/4LH_standard_ | 1.03±0.21 | 0.92±0.17 | -2.24 | 0.029 |
| 1/4HL_standard_ | 0.99±0.23 | 0.95±0.16 | -0.85 | 0.399 |
| 1/8LL_mean_ | 0.87±0.29 | 1.05±0.17 | 3.05 | 0.003 |
| 1/8LH_mean_ | -0.61±0.72 | -0.82±0.65 | -1.18 | 0.243 |
| 1/8HL_mean_ | 0.03±1.04 | -0.47±0.85 | -2.06 | 0.044 |
| 1/8LL_standard_ | 0.96±0.21 | 0.99±0.15 | 0.82 | 0.416 |
| 1/8LH_standard_ | 0.96±0.12 | 1.01±0.1 | 1.67 | 0.100 |
| 1/8HL_standard_ | 0.97±0.12 | 1±0.1 | 0.96 | 0.342 |
| 1/16LL_mean_ | 0.87±0.28 | 1.05±0.18 | 3.11 | 0.003 |
| 1/16LH_mean_ | 0.05±1.14 | 0.23±0.83 | 0.71 | 0.484 |
| 1/16HL_mean_ | -0.41±0.91 | -0.63±0.79 | -0.99 | 0.327 |
| 1/16LL_standard_ | 0.9±0.25 | 1.04±0.14 | 2.53 | 0.014 |
| 1/16LH_standard_ | 0.94±0.14 | 1.03±0.11 | 2.88 | 0.006 |
| 1/16HL_standard_ | 0.93±0.14 | 1.03±0.12 | 2.70 | 0.009 |
| Third order wavelet | | | | |
| 1/2LL_mean_ | 0.87±0.28 | 1.05±0.16 | 3.13 | 0.003 |
| 1/2LH_mean_ | -0.03±1.15 | 0.01±0.84 | 0.15 | 0.878 |
| 1/2HL_mean_ | 0.59±1.02 | 0.43±0.68 | -0.70 | 0.488 |
| 1/2LL_standard_ | 1.04±0.34 | 0.87±0.16 | -2.48 | 0.016 |
| 1/2LH_standard_ | 1.02±0.49 | 0.81±0.16 | -2.24 | 0.029 |
| 1/2HL_standard_ | 0.96±0.37 | 0.92±0.24 | -0.48 | 0.632 |
| 1/4LL_mean_ | 0.88±0.29 | 1.04±0.15 | 2.77 | 0.007 |
| 1/4LH_mean_ | 0.16±0.93 | 0.06±1.07 | -0.40 | 0.691 |
| 1/4HL_mean_ | 0.35±0.89 | 0.18±1.04 | -0.68 | 0.498 |
| 1/4LL_standard_ | 1.04±0.24 | 0.89±0.18 | -2.74 | 0.008 |
| 1/4LH_standard_ | 1.05±0.27 | 0.87±0.17 | -3.10 | 0.003 |
| 1/4HL_standard_ | 1.03±0.19 | 0.92±0.17 | -2.39 | 0.020 |
| 1/8LL_mean_ | 0.86±0.29 | 1.05±0.18 | 3.08 | 0.003 |
| 1/8LH_mean_ | -0.4±0.89 | -0.18±1.03 | 0.87 | 0.391 |
| 1/8HL_mean_ | -0.47±0.66 | -1.01±0.55 | -3.45 | 0.001 |
| 1/8LL_standard_ | 0.91±0.26 | 1.02±0.14 | 2.04 | 0.046 |
| 1/8LH_standard_ | 0.94±0.16 | 1.02±0.14 | 2.07 | 0.043 |
| 1/8HL_standard_ | 0.95±0.17 | 1.01±0.13 | 1.47 | 0.148 |
| 1/16LL_mean_ | 0.87±0.28 | 1.05±0.18 | 3.13 | 0.003 |
| 1/16LH_mean_ | -0.27±0.96 | -0.16±1.01 | 0.44 | 0.665 |
| 1/16HL_mean_ | -0.44±0.69 | -1±0.57 | -3.40 | 0.001 |
| 1/16LL_standard_ | 0.89±0.27 | 1.04±0.15 | 2.69 | 0.009 |
| 1/16LH_standard_ | 0.93±0.18 | 1.02±0.15 | 2.11 | 0.040 |
| 1/16HL_standard_ | 0.95±0.19 | 1.01±0.15 | 1.38 | 0.172 |

**Supplementary table 1.** All ADC characteristic values (histogram, texture, wavelet) and the t value and p value after t test.

Note: The part marked in red is the features finally selected by lasso after dimensionality reduction. The highlighted part is the ADC characteristic of the PRs group higher than the normal group; the unmarked part is the ADC characteristic of the PRs group lower than the normal group.

For ADC histogram characteristics, except for ADCmax, Skewness, Kurtosis, Entropy, and variance, the ADC histogram characteristics of the PRs group are lower than the normal group, and the differences are statistically significant (P <0.05). For texture features, the values of contrast and dissimilarity in the PRs group are lower than those in the normal group. However, for the values of homogeneity, ASM, and energy, the PRs are higher than the normal group, and the differences are statistically significant.

We found that the ADC histogram characteristics of the PRs group were relatively lower than that of the normal group, which may be related to hypoxia and abnormal brain development [17] [27]. The texture characteristics and wavelet characteristics were also significantly different in our two groups.
