# Supplementary material for: Machine Learning Models on ADC Features to Assess Brain Changes of Children With Pierre Robin Sequence
Source: Front Neurol. 2021 Mar 4;12:580440. doi: 10.3389/fneur.2021.580440 (PMC7969993; doi:10.3389/fneur.2021.580440)
Supplement: Supplementary file 2 [file Data_Sheet_1.PDF]

```
‘----------------This module is for feature extracting--------------'
import os
import time
import FeatureAnalysis as FA
import numpy as np
import preprocess as pp
import warnings
warnings.filterwarnings("ignore")

#The directory path of data.
dirpath =""




#dirpath =""
OriginData,T1_spacing = pp.loadData(dirpath,mode='1.5')


# show the ROI of slice in the Patient ID given
index = 0 #the patient ID
slice_index = 7 #the slice index of the patient ID given.
pp.showROI(OriginData,index,slice_index)


PatientNum = OriginData.shape[0]
ModelNum = OriginData.shape[1]
SliceNum = OriginData.shape[4]
Rows = OriginData.shape[2]
Columns = OriginData.shape[3]
paraTotal = []
for index in range(PatientNum):
    print('processing......', index, '/', PatientNum)
    paraADC = []
    paraT1 = []
    paraT2 = []
    ROIpixels = 0
    for s in range(SliceNum):
        T1ROI = np.zeros((Rows,Columns))
        T2ROI = np.zeros((Rows,Columns))
        ADCROI = np.zeros((Rows,Columns))
        ADC = OriginData[index,0,:,:,s]
        T1 = OriginData[index, 1, :, :, s]
        T2 = OriginData[index, 2, :, :, s]
        ROI = OriginData[index, 3, :, :, s]
```

```
            for i in range(Rows):
                for j in range(Columns):
                    if(ROI[i,j]):
                        ROIpixels = ROIpixels+1
                        T1ROI[i,j] = T1[i,j]
                        T2ROI[i, j] = T2[i, j]
                        ADCROI[i, j] = ADC[i, j]
            if(not(np.all(np.equal(T1ROI,0)))):
                try:
                    paralist = FA.HistogramAnalysis(T1ROI) + FA.TextureAnalysis(np.uint16(T1ROI))
+ tuple(FA.Wavelet(np.uint16(T1ROI)))
                    paralist = np.float64(np.asanyarray(paralist))
                    paraT1.append(paralist)
                    paralist = FA.HistogramAnalysis(T2ROI) + FA.TextureAnalysis(np.uint16(T2ROI))
+ tuple(FA.Wavelet(np.uint16(T2ROI)))
                    paralist = np.float64(np.asanyarray(paralist))
                    paraT2.append(paralist)
                    paralist = FA.HistogramAnalysis(ADCROI) +
FA.TextureAnalysis(np.uint16(ADCROI))+ tuple(FA.Wavelet(np.uint16(ADCROI)))
                    paralist = np.float64(np.asanyarray(paralist))
                    paraADC.append(paralist)
                except:
                    print('Analysis failed!')
        paraT1 = np.asanyarray(paraT1).mean(axis=0)
        paraT2 = np.asanyarray(paraT2).mean(axis=0)
        paraADC = np.asanyarray(paraADC).mean(axis=0)
        VolumnSize = T1_spacing*ROIpixels # mm*mm*mm
        paraADC = list(paraADC)
        paraADC.append(VolumnSize)
        para = np.asanyarray(list(paraADC) + list(paraT1)+ list(paraT2))
        paraTotal.append(para)

        # The parameters:
        # Histogram parameters:
        # 01Mean,02Min,03Max,04Skewness, 05Kurtosis, 06Entropy, 07Mode_count,
08Mode_Value,
        # 09Variance,10Percentile_10, 11Percentile_20, 12Percentile_30, 13Percentile_40,
        # 14Percentile_50,15Percentile_60, 16Percentile_70, 17Percentile_80, 18Percentile_90,
        # 19Percentile_15,20Percentile_25, 21Percentile_35, 22Percentile_45, 23Percentile_55,
        # 24Percentile_65,25Percentile_75, 26Percentile_85, 27Percentile_95
        # Volumn size:
        # 28VolumnSize
        # Texture parameters:
        # 29contrast,30dissimilarity,31homogeneity,32ASM,33energy,34correlation
```

```
import pandas as pd
data_df = pd.DataFrame(paraTotal)
writer = pd.ExcelWriter(os.path.join(dirpath,time.strftime('%Y%m%d%H%M%S',
time.localtime(time.time()))+'para' + '.xlsx'))
data_df.to_excel(writer, 'page_1', float_format='%.5f')     # float_format
writer.save()
```
